# Alternate Cervical Venous Access Sites for Implantable Port Catheters: Experience at a Single Quaternary Care Institution

**DOI:** 10.1007/s00270-022-03306-9

**Published:** 2022-12-12

**Authors:** Frank K. Liou, Patrick Y. Kim, S. Paran Yap, Abdullah Khan, Sandra Taylor, Rex Pillai, Eric King, Amol Shah, R. Torrance Andrews, Catherine T. Vu, Roger E. Goldman

**Affiliations:** 1 Davis Medical Center. Department of Radiology, University of California, 4860 Y Street, Suite 3100, Sacramento, CA 95817 USA; 2 Davis Medical Center, Clinical and Translational Science Center, University of California, Sacramento, CA USA

**Keywords:** Central venous access, Central vein occlusion, Port complications

## Abstract

**Introduction:**

Clinical outcomes of implantable port catheters (IPCs) placed via alternative veins such as the external jugular and cervical collaterals have not been well established. This investigation evaluates the short- and long-term outcomes of IPCs inserted via alternate cervical veins (ACV) compared to traditionally inserted IPCs via the internal jugular vein (IJV).

**Materials and Methods:**

A total of 24 patients who received an IPC between 2010 and 2020 via an ACV—defined as the external jugular vein, superficial cervical vein, or unnamed collateral veins—were identified. Based on power analysis, a matched control group of 72 patients who received IPCs via the IJV was identified. Non-inferiority analysis for port complications was performed between the two groups based on the selected non-inferiority margin of 20%. Secondary end points included complication-free survival and comparison of complications by the time at which they occurred.

**Results:**

ACV access was non-inferior to traditional access for overall complications. Alternate access resulted in fewer complications than traditional access with an estimated reduction of − 7.0% [95% CI − 23.6%, 39.7%]. There was no significant difference in peri-procedural and post-procedural complications between the two groups. Complication-free survival was also equivalent between the two groups.

**Conclusion:**

IPC placement via ACVs was non-inferior to IPCs placed via traditional access through the IJV. When abnormal pathology obviates the use of IJV access, other cervical veins may be considered prior to seeking alternate locations such as femoral, translumbar, inferior vena cava, and hepatic veins.

## Introduction

Percutaneous implantable port catheters (IPCs) have become the primary venous access by which patients may receive long-term medication infusions [1, 2]. With image-guided percutaneous placement of IPCs established as the standard of practice over unguided placement techniques [3–5], operators are able to evaluate for and select the optimal venous access sites for these devices [6]. When the preferred internal jugular vein (IJV) is unavailable, most commonly due to stenosis or obstruction, other cervical veins offer potential alternatives.

There is currently limited evidence directly documenting the safety and efficacy of IPCs inserted via these alternate cervical access sites. Kato et al. and Lorio et al. have both reported on the use of the external jugular vein (EJV) for IPC placement. However, these prior studies are limited by a lack of a control group for comparison and the inclusion of surgically inserted EJV IPCs via cut-down technique, respectively [7, 8]. Additionally, neither group included the use of thyrocervical collateral veins.

While the use of these alternative sites has been incompletely investigated with respect to IPC placement, anecdotal publications and retrospective studies have evaluated placement of tunneled and non-tunneled central venous catheters in alternate cervical veins. Forauer et al. reported freedom from peri-procedural complications in a series of ten hemodialysis catheter placements in eight patients, with 100% primary patency [9]. Funaki et al. reported a retrospective series of patients with documented occluded internal jugular veins with nine successful hemodialysis catheter placements in thyrocervical collateral veins [10]. Cho et al. reported a retrospective series of twenty-three patients undergoing tunneled hemodialysis catheter placement via the right external jugular vein (EJV) with a 96% technical success rate and an overall complication rate of 0.22 per 100 catheter days [11].

The current Kidney Disease Outcome Quality Initiative (KDOQI) clinical practice guidelines recommend the IJV to be utilized first with the EJV and femoral veins following in order of preference for the insertion of tunneled dialysis catheters [12]. Given these prior successful outcomes with external jugular and thyrocervical collateral venous access for tunneled hemodialysis CVCs, we hypothesized that similar safe outcomes would be expected in dedicated analysis of patients receiving IPCs via alternate cervical access sites.

## Materials and Methods

Institutional review board exemption was received for this single-center retrospective case control study. Study cohorts were identified for inclusion by query of an institutional radiologic database for all patients who underwent an IPC placement procedure between January of 2010 and December of 2020. All procedures were performed using ultrasound-guided access, followed by fluoroscopic guidance for guidewire manipulation and component placement. Patients were excluded if the venous access location could not be definitively identified via the imaging or medical record. Patients were also excluded if they received IPCs via the subclavian, common femoral, or femoral vein. Alternative venous access sites were defined as EJV, superficial cervical vein, or an unnamed collateral vein within the neck. A consecutive cohort of 24 patients receiving IPCs via an alternate cervical vein was identified. Based on power analysis, a three-to-one control group of 72 patients receiving IPCs via the standard IJV (i.e., traditional access) was identified, matched for age, gender, and indication for port insertion (Table 1).

**Table 1:** Ported catheter placement indication

	Non-traditional access (*N* = 24)	Traditional access (*N* = 72)
*Ports for oncologic therapy*	20	59
Hematologic	6	19
Breast	4	12
Gynecologic	4	12
Colorectal	3	10
Pancreatic	1	3
Neurologic	1	0
Dermatologic	1	3
*Ports for non-oncologic purposes*	4	13
Sickle cell anemia	1	4
Difficult peripheral vascular access	1	4
Autoimmune	1	1
Post-transplant requiring access	1	4

Data were collected through review of the patients’ electronic medical records, procedural images, and post-procedural images. Patient characteristics included age, gender, BMI, diabetes diagnosis, cancer diagnosis, cancer stage, ECOG status, and anticoagulant use at the time of port insertion. Intraprocedural and post-procedural data, including technical success, and complications were recorded. Complications were defined per the Society of Interventional Radiology Quality Improvement Guidelines as pneumothorax, hemothorax, hematoma, perforation, air embolism, wound dehiscence, sepsis, thrombosis, or port malfunction requiring intervention [6].

The primary outcome evaluated was the overall complication rate within the alternate access and control groups. Port complications were stratified by time after insertion, into peri-procedural (within 24 h post-procedure), early (between 1 and 30 days post-procedure), and late (greater than 30 days post-procedure). Secondary outcomes evaluated were complications stratified by time of insertion, requirements for additional complex insertion techniques, and differences in IPC sizes were compared between the two groups.

## Statistical Analysis

Prior to analysis, data integrity was assessed by generating univariate and bivariate contingency tables for categorical variables and for numeric variables graphically and through summary statistics (mean, quartiles, range) to identify unusual or extreme values. Categorical variables are summarized as counts and proportions; quantitative variables as mean ± standard deviation (SD). The size of the control group was determined to test if the complication rate among alternate access patients was non-inferior to the control group. Using a two-sample proportion test and assuming a complication rate of 10% [13–15], 24 patients in the alternate venous access group and 72 control patients provide 88% power at a significance level of 5% to reject the null hypothesis that alternate access is inferior to traditional access for a non-inferiority margin of 20%. The non-inferiority margin of 20% was selected based upon the sample size of the alternate access group and statistical power. Because of the relatively small and fixed number of alternate access patients, even using a 3:1 ratio of traditional/alternate access patients, statistical power was only adequate for a relatively large non-inferiority margin of 20%. Therefore, we report estimated differences and 95% confidence intervals in addition to results of the non-inferiority test.

Continuity corrected *Z*-test for proportions using the pooled standard deviation was performed to determine whether alternate neck access was non-inferior to traditional IJV access in terms of complication rates within the established 20% margin. Hypothesis tests were evaluated at a significance level of 0.05 and were two-sided except for tests of non-inferiority which were one-sided tests. Categorical variables were compared using Fisher’s exact test, and continuous variables were compared using Student’s two-sample *t* test. Statistical analyses were conducted using R version 4.0.3 and SPSS 27.

## Results

All patients receiving IPCs had absolute neutrophil counts > 1500, platelet count > 50,000, and INR < 1.8 prior to insertion. Baseline demographics data of IPC recipients are presented in Table 2. The alternate venous access group had a significantly higher incidence of patients with prior indwelling catheters (*P* = 0.002) and percentage with high-grade internal jugular vein stenosis, as defined by the operator at the time of port placement (*P* < 0.001). There was otherwise no statistically significant difference between the two groups in terms of age, gender, BMI, diabetes incidence, cancer diagnosis, cancer stage, ECOG status, and anticoagulant use at the time of port insertion.

**Table 2:** Patient demographics

	Non-IJ group (*N* = 24)	IJ group (*N* = 72)	*P*-value
Average BMI	27.6 ± 7.7	26.1 ± 6.5.8	0.316
Diabetes	20.8% (20/24)	23.6% (17/72)	> 0.999
Percent requiring chemotherapy	83.3% (20/24)	81.9% (59/72)	> 0.990
Cancer stage 1	5.0% (1/20)	6.8% (4/59)	> 0.999
Cancer stage 2	25.0% (5/20)	28.8% (17/59)	> 0.999
Cancer stage 3	50.0% (10/20)	40.7% (24/59)	0.602
Cancer stage 4	20.0% (4/20)	22.0% (13/59)	> 0.999
Cancer stage unknown	0.0% (0/20)	1.7% (1/59)	> 0.999
Average ECOG status	0.9 ± 0.5	0.7 ± 0.8	0.334
Percent on anticoagulation	20.8% (5/24)	9.1% (7/72)	0.168
Percent on antiplatelet therapy	8.3% (2/24)	8.3 (6/72)	> 0.999
Percent with previous indwelling catheter*	50.0% (12/24)	16.7% (12/72)	0.002
Median indwell time of prior catheter (days)	1595	343	–
Percent with high-grade IJ stenosis^#^	65.2% (15/23)	0.0% (0/72)	< 0.001

*Includes venous access ports and tunneled dialysis catheters

^#^As defined by the primary operator at the time of port placement

Within the alternative venous access cohort, IPCs were placed via the external jugular vein (*n* = 20), an unnamed cervical collateral vein (*n* = 3), or the superficial cervical vein (*n* = 1). Of the 24 patients who received IPC placement via an alternative access, none required advanced techniques such as venoplasty or stenting during the procedure. The indications for alternate venous access were severe stenosis or occlusion of the bilateral internal jugular veins as evidenced by ultrasound and/or venography (*n* = 16), obstruction by the presence of an additional central venous catheter (*n* = 1), bilateral internal jugular veins not reliably identified sonographically (*n* = 3), and reason(s) not documented in the procedural note and stored imaging on PACS (*n* = 4). Major peri-procedural complications occurred in 2.8% of patients in the traditional access group. The major peri-procedural complications sustained by the traditional access group included pneumothorax requiring chest-tube placement and wound dehiscence requiring a new IPC within 24 hours following initial port placement. No major peri-procedural complications were sustained by patients within the alternative venous access group. One minor complication occurred within the non-traditional access group, in the form of intraprocedural pain prompting the use of general anesthesia. Alternate access resulted in fewer complications than traditional access (8.3% [2 out of 24] vs 15.3% [11 out of 72]) with an estimated reduction of − 7.0% [95% CI − 23.6%, 39.7%]. Based on the selected non-inferiority margin, alternate access was found to be non-inferior to traditional access in terms of overall complications (*P* < 0.001).

**Fig. 1:**
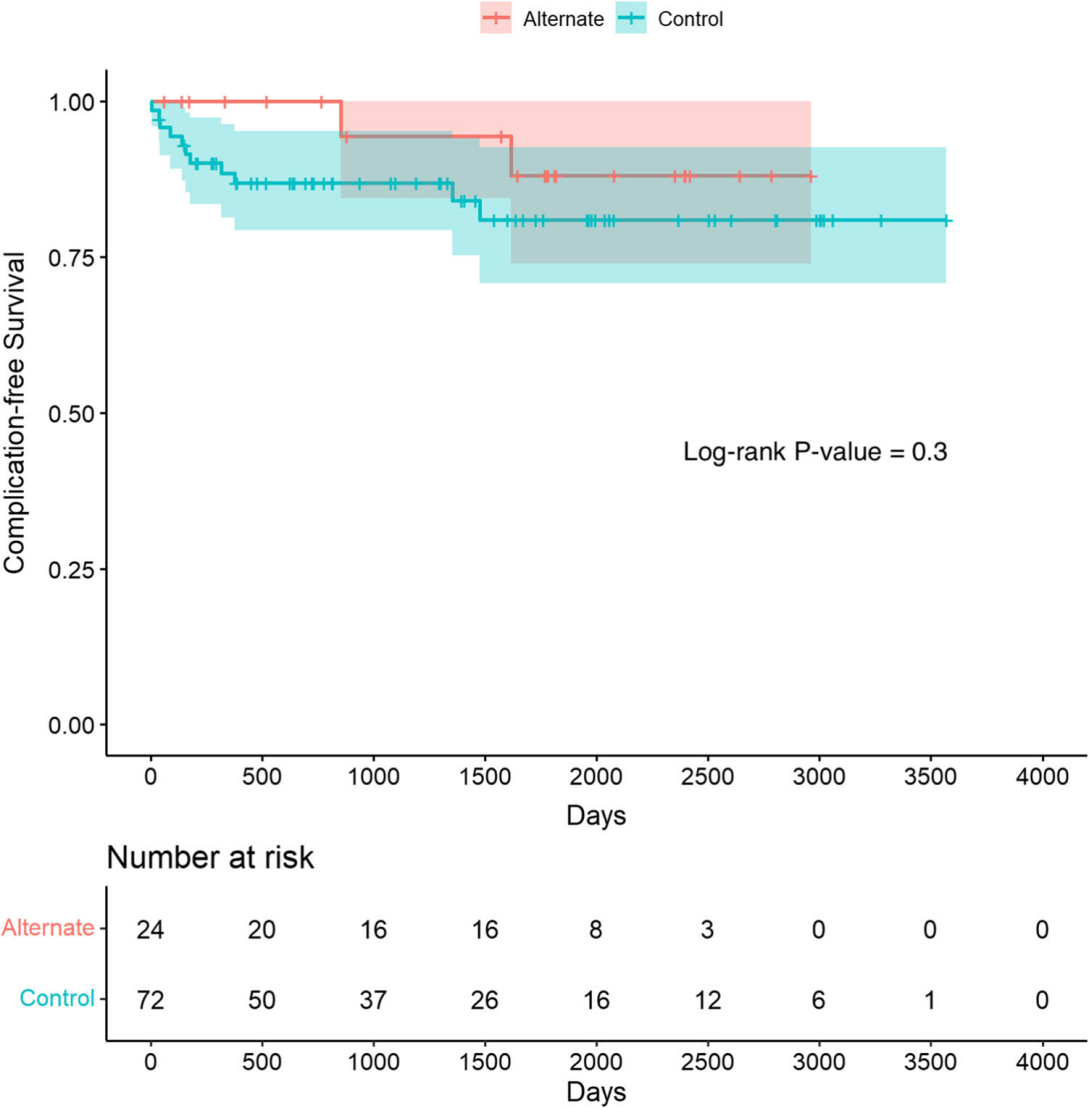
Kaplan–Meier estimates for freedom from major complication

Complication-free catheter days was similar between the non-traditional and traditional access groups (1564.9 ± 892.6 vs 1252.5 ± 985.7; *P* = 0.174). Incidence of early and late complications was also comparable between the two groups (Table 3). Complications included infection, port thrombosis requiring revision, and port site bleeding (Table 4). Port infection and/or sepsis were the most common complication in both groups. Comparison of complication-free survival between the two groups using Kaplan–Meier analysis also found no significant difference between the two groups (*P* = 0.3) (Fig. 1). There was also no significant difference in French size of catheters used between the alternative access and traditional access groups (8.6 ± 1.2 Fr vs 8.4 ± 1.0 Fr; *P* = 0.422).

**Table 3:** Port-related major complications grouped by time from procedure

	Non-traditional access (*N* = 24)	Traditional access (*N* = 72)	*P*-value
Total complications	8.3% (2/24)	15.3% (11/72)	0.507
Immediate (procedural)	0% * (0/24)	2.8% (2/72)	> 0.999
Delayed	8.3% (2/24)	12.5% (9/72)	> 0.999
Early	0% (0/2)	10.0% (1/10)	> 0.999
Late	100% (2/2)	90.0% (9/10)	> 0.999

*One minor complication (pain requiring general anesthesia)

**Table 4:** Port-related complications by type

Complication type	Non-traditional access (*N* = 2)	Traditional access (*N* = 9)
Infection	100.0% (2/2)	55.6% (5/9)
Port thrombosis requiring revision	0.0% (0/2)	33.3% (3/9)
Port site bleeding	0.0% (0/2)	11.1% (1/9)

## Discussion

We hypothesized that the outcomes for alternative venous access sites would mirror the acceptable safety profile demonstrated in the outcomes of external jugular vein access for tunneled hemodialysis catheters [11]. The results of our study demonstrate non-inferiority of alternate cervical venous access IPCs to traditional access IPCs both in the peri-procedure and long-term post-procedure period. The groups had comparable complication-free survival post-procedurally. Further stratification also demonstrates similar complication rates between the two groups, with port infections and sepsis being the most frequent complication. Notably, most of these infectious complications occurred greater than 30 days following the port insertion, suggesting a complication of port access or infusion technique as an inciting factor as opposed to the port insertion technique. The data in the current study compare favorably to the published literature with respect to complication rates which have been reported between 7.2 and 19.0% [13–15]. Similar to our findings, port infections and sepsis also accounted for the majority of the port-related complications reported in these publications, occurring in 2.5–6.9% of the studied cohorts.

Placement of alternative access IPCs also did not require advanced techniques involving central venous reconstruction, nor did it require operators to use smaller French size catheters. This suggests that the non-inferior outcomes of the alternate access group were achieved without techniques that would otherwise not be used during routine IJV IPC insertions. The use of alternative venous access sites also did not demonstrate an increased risk of peri-procedural or long-term complications when compared to IPCs placed via the internal jugular veins. The similar outcomes are hypothesized to be due to the similar course of the catheter inserted via these alternate access sites both in the tunneled extravascular section and the intravascular section, with the exception of the short distance within the superficial non-traditional collateral vein when compared to traditional catheters inserted via the IJV. As such, there appears to be no significant difference in either total complication-free survival or complication type.

The present study has several limitations. The study is limited by the potential for error in the medical record and failure to capture patient care outside of the institution. Non-standard cervical access procedures could only be identified based on the presence of clear procedural documentation. The analysis may therefore have excluded additional such cases that might have occurred. It is also possible that non-standard access might have been attempted unsuccessfully without identification by the study protocol. Furthermore, the relatively small sample size of our alternate cervical access population and low complication rate limited the minimum non-inferiority margin that could be evaluated with sufficient statistical power and yielded low statistical power of the time-to-event analysis. Additionally, within the alternate cervical access population, the majority of patients underwent IPC placement via the EJV, with only 16.7% receiving IPCs via alternate cervical veins. The study is underpowered to evaluate the exclusive use of unnamed cervical collaterals and superficial cervical veins. Thus, the exclusive approach accessing via these veins may not generalize to the outcomes of the analyzed alternate access cohort including both EJV and cervical collaterals. Lastly, noting the patient population was recruited from a single center, the techniques of ported catheter insertion and devices, while considered standard interventional radiology practice, may not generalize to all operators.

## Conclusion

In our single-center experience, the use of alternate cervical venous access sites for IPCs placement is non-inferior to IPCs placed via traditional access through the IJV. When abnormal pathology obviates the use of IJV access, other smaller veins of the neck may be considered for access prior to seeking alternate locations such as femoral, translumbar inferior vena cava, hepatic veins, and intercostal veins. Future studies to determine the efficacy of alternate cervical venous access sites vs IJV access for IPC placement may strengthen the results of the current investigation.
